# Photocatalytic Antibacterial Effects Are Maintained on Resin-Based TiO_2_ Nanocomposites after Cessation of UV Irradiation

**DOI:** 10.1371/journal.pone.0075929

**Published:** 2013-10-17

**Authors:** Yanling Cai, Maria Strømme, Ken Welch

**Affiliations:** Division for Nanotechnology and Functional Materials, Department of Engineering Sciences, The Ångström Laboratory, Uppsala University, Uppsala, Sweden; RMIT University, Australia

## Abstract

Photocatalysis induced by TiO_2_ and UV light constitutes a decontamination and antibacterial strategy utilized in many applications including self-cleaning environmental surfaces, water and air treatment. The present work reveals that antibacterial effects induced by photocatalysis can be maintained even after the cessation of UV irradiation. We show that resin-based composites containing 20% TiO_2_ nanoparticles continue to provide a pronounced antibacterial effect against the pathogens *Escherichia coli*, *Staphylococcus epidermidis*, *Streptococcus pyogenes*, *Streptococcus mutans and Enterococcus faecalis* for up to two hours post UV. For biomaterials or implant coatings, where direct UV illumination is not feasible, a prolonged antibacterial effect after the cessation of the illumination would offer new unexplored treatment possibilities.

## Introduction

Titanium dioxide (TiO_2_) is a photocatalyst that has been investigated for a wide variety of applications such as the decontamination of water [1] and air [2] as well as for self-cleaning surfaces [3]. When irradiated with UV-A light (λ<385 nm for the anatase form of TiO_2_), crystalline TiO_2_ is activated and electron (e^−^) – hole (h^+^) pairs are formed. The excited electron e^−^ can be trapped at surface Ti^IV^ sites of TiO_2_ to form the reduced Ti^III^ sites (Eq. 1), which in turn can react with O_2_ to generate superoxide radicals (O_2_^•−^) (Eq. 2). Conversely, the positive holes generate hydroxyl radicals (•OH) through the reaction with water or hydroxyl ions on the hydrated metal oxide (Eqs. 3 and 4). Further reactions can lead to the formation of hydrogen peroxide (H_2_O_2_). Reactive oxidative species (ROS), including •OH, O_2_^•−^ and H_2_O_2_, are considered as the main products of photocatalytic reaction [4].

TiO_2_ induced photocatalysis has been investigated for applications in various antimicrobial materials [5]–[10]. The antibacterial mechanism is a result of the reaction between the ROS and bacterial structural components, like the cell wall and cell membrane [11], [12]. Particularly, unsaturated phosphate lipids in the cell membrane are the most sensitive targets for the ROS attack and lipid peroxidation is considered to be the lethal mode of action in the photocatalytic antibacterial process [13], [14].

TiO_2_ nanoparticles are widely used as photocatalysts because of their high photocatalytic activity, mainly due to their high specific surface area [15]. A number of polymer-TiO_2_ composites have been developed to take advantage of their combined synergetic properties. For example, TiO_2_ nanoparticles embedded in polysulfone [16] and polyethersulfone [17] membranes improve the water affinity, mechanical strength and anti-fouling ability of the membranes. Bactericidal effect under UV irradiation has also been shown in composite materials through the addition of TiO_2_ nanomaterials to polymers like polyurethane [18], polypropylene [19], [20], aromatic polyamide [21], cellulose [22] and polystyrene [23]. We have previously demonstrated that a resin-TiO_2_ nanocomposite prepared through the addition of TiO_2_ nanoparticles to a dental adhesive resin material endows the nanocomposite with antibacterial and bioactive properties without affecting the functional bonding strength of the adhesive [24].

The photocatalytic effect requires the application of UV light which is both an advantage; since the effect can be induced on demand, and a disadvantage; since UV illumination is not always possible or practical. For certain biomaterials applications a prolonged antibacterial effect after the cessation of UV irradiation would offer benefits. In this work, we present a novel property of a resin-TiO_2_ nanocomposite in which an antibacterial effect is shown to be present for a significant time following UV irradiation. This post-UV antibacterial property of the resin-TiO_2_ samples was tested on five bacterial strains including *Escherichia Coli*, *Staphylococcus epidermidis*, *Streptococcus pyogenes*, *Streptococcus mutans and Enterococcus faecalis*. Additionally, the tendency for *S. epidermidis* adhesion on the UV-treated resin-TiO_2_ samples was investigated.

## Materials and Methods

### Bacterial strains

*Escherichia coli* (DH5α) and four pathogenic bacterial strains: *Staphylococcus epidermidis* (CCUG18000A), *Streptococcus pyogenes* (BM137), *Streptococcus mutans* (UA159), *Enterococcus faecalis* (JH2-2) were activated in Brain Heart Infusion (BHI) broth and cultured at 37°C to late log phase. The bacteria were collected by centrifugation (4000 rpm, 10 min, EBA 30 centrifuge, Hettich, Tuttlingen, Germany) and then re-suspended in sterile PBS (phosphate buffered saline, Sigma-Aldrich, Germany). Bacterial density was adjusted to 10^9^ CFU/mL for all strains using optical density measurements.

### Resin-TiO_2_ nanocomposite

The resin consists of two types of monomer, 2, 2-bis [4-(2-hydroxy-3- methacryloxypropoxy) phenyl-propane (BisGMA, Polysciences Europe GmbH, Eppelheim, Germany) and 2-hydroxyethyl methacrylate (HEMA, Sigma-Aldrich, Schnelldorf, Germany) in a 55/45 wt/wt ratio. Photoinitiator and coinitiators were added as follows: 0.5 mol% camphorquinone (CQ); 0.5 mol% 2-(dimethylamino) ethyl methacrylate (DMAEMA); 0.5 mol% ethyl-4-(dimethylamino) benzoate (EDMAB); and 1 wt% diphenyliodoniumhexafluorophosphate (DPIHP) (all from Sigma-Aldrich, Steinheim, Germany).

The resin-TiO_2_ nanocomposite was made by mixing 20 wt% of photocatalytic TiO_2_ nanoparticles (P25, Evonik Industries (previously Degussa) AG, Germany) in the resin. P25 TiO_2_ nanoparticles are comprised of anatase and rutile crystalline phases in a ratio of about 3∶1 [25]. It has previously been shown [24] that the composition of 20 wt% P25 TiO_2_ nanoparticles in the resin matrix provides optimal antibacterial effect without negatively affecting its functional performance as a dental adhesive. The container with the resin-TiO_2_ mixture was sonicated for 1 hour to minimize nanoparticle aggregation. The resin-TiO_2_ mixture was then cast in Teflon molds (diameter 8 mm, thickness 1 mm) and cured under 460 nm light (BlueLEX GT1200, Monitex, Taiwan) for 30 s under N_2_ flow. Scanning Electron Microscopy (SEM, LEO 1550, Zeiss) images of resin disks with and without TiO_2_ nanoparticles were recorded after sputter coating with gold/palladium (Polaron SC7640, Thermo VG Scientific). Additionally, the resin-TiO_2_ nanocomposite disks were characterized through X-ray diffraction (XRD, D-5000 X-ray diffractometer, Siemens).

### UV pre-treatment of resin-TiO_2_ disks

Three groups of resin-TiO_2_ sample disks were prepared: resin-TiO_2_ disks without UV irradiation treatment (resin-TiO_2_ control disks); resin-TiO_2_ disks pre-treated with UV irradiation under ambient conditions (post-UV dry disks) and resin-TiO_2_ disks pre-treated with UV irradiation under aqueous conditions (post-UV wet disks).

To treat the resin-TiO_2_ disks with UV irradiation in ambient conditions, disks were placed in a petri dish covered with its transparent lid and irradiated with a UV-A diode (peak wavelength at 365 nm, NSCU033B (T), Nichia, Japan) for 1 hour at an intensity of 10 mW/cm^2^ (UV light meter, UV-340, Lutron). To treat the resin-TiO_2_ disks with UV irradiation in aqueous environment, 50 µL of deionized water was spread on the surface of each disk prior to UV irradiation. The disks were subsequently placed in a petri dish with its transparent lid and irradiated with the UV-A diode for 1 hour (10 mW/cm^2^). After the UV treatment, the water drops were collected from the surface of the disks by pipetting and transferred to individual wells in a 48-well plate.

### Post-UV antibacterial tests

Immediately after the UV treatment, the antibacterial properties of the resin-TiO_2_ control disks, post-UV dry disks, post-UV wet disks and water droplets extracted from the surfaces of the post-UV wet disks were investigated. All antibacterial tests were performed in triplicate for each bacterial strain.

For antibacterial testing of the disks, 10 µL of bacterial suspension were spread on each resin-TiO_2_ disk. Disks with bacteria were placed in a petri dish, covered with its lid and incubated for 15 minutes at room temperature. Individual disks were subsequently transferred into wells in a 48-well plate with 300 µL sterile PBS in each well and orbital shaking at 500 rpm for 2 min was applied to re-suspend the bacteria from the resin-TiO_2_ disks. The disks were then removed from the wells.

For antibacterial testing of the water drops from the post-UV wet disks, 10 µL of bacterial suspension were mixed into the water drop by pipetting and incubated for 15 minutes at room temperature. Following the incubation, 300 µL sterile PBS were added to the wells containing water drops with bacteria and orbital shaking at 500 rpm for 2 min was applied to re-suspend the bacteria from the bottom of the wells.

Bacterial viability of the samples was quantified using a metabolic assay incorporating the indicator resazurin. One milliliter of BHI broth with resazurin (1.25 µg/mL) was added to each well containing a sample of bacterial suspension. Concurrently, a dilution series of bacteria suspension of the corresponding bacterial strain with known bacteria concentration was measured to produce a standard curve for quantification of the test samples. The assays were incubated at 37°C for 3 hours and the production of resorufin, indicating the bacterial metabolic activity, was measured with a fluorescent multiplate reader (excitation at 530 nm, emission at 590 nm, Tecan F200). The number of viable bacteria in each test was determined with aid of the standard curve. Bacteria from the resin-TiO_2_ control disks were utilized to define 100% bacterial viability.

### UV pre-treatment of pure resin disks and TiO_2_ nanoparticles

In order to determine if the post-UV antibacterial effect was only observed with the combination of TiO_2_ nanoparticles and resin, antibacterial tests were also performed using TiO_2_ nanoparticles and disks comprised of the resin polymer without TiO_2_ nanoparticles. Both the UV pre-treatment (1 hour, 10 mW/cm^2^) in ambient and aqueous environments and the post-UV antibacterial testing were performed as detailed above. Only *S. epidermidis* was used in these control tests.

### Bacterial adhesion tests

The post-UV antibacterial effect was also tested on the tendency for *S. epidermidis* adhesion on the disk surfaces as well as the viability of the adhered bacteria. Gram-positive *S. epidermidis* (CCUG 18000A) is a skin flora species and a common cause for infections associated with implant or biomedical devices [26]. Adhesion testing was performed on both post-UV dry disks and post-UV wet disks following a delay period of 0, 15, 30, 60 or 120 minutes after the UV pre-treatment described above. Four disks were used for each delay period and disk type. Additionally, two control groups consisting of four resin-TiO_2_ disks without UV pre-treatment were included in the adhesion testing. Control Group 1 were tested in the same manner as the post-UV dry and post-UV wet disks, while control Group 2 received a UV dose of 36 J/cm^2^ during the adhesion testing (10 mW/cm^2^ for 1 hour).

Adhesion testing was performed through the incubation of the disks in a 10 mL bacterial suspension in a well of a 6-well plate under shear forces generated with the help of an orbital shaker. The bacterial suspension for adhesion testing was prepared by suspending *S. epidermidis* in sterile PBS with a concentration of 10^8^ CFU/mL. The resin-TiO_2_ disks were secured on the bottom of the well with the aid of a rubber mold such that all disks were located at the same distance from the center of the well and therefore experienced the same shear forces during the culturing. The 6-well plate containing the disks was fixed on an orbital shaking incubator (Talboys, Troemner, USA) set to 37°C. The culturing process consisted of 30 min static incubation to allow for initial adhesion followed by 30 min incubation with 100 rpm orbital shaking. After the adhesion culturing, the resin-TiO_2_ disks were removed from the bacterial suspension and rinsed gently with sterile PBS to wash away non-adherent bacteria.

One disk from each of the control disks, the post-UV dry disks and the post-UV wet disks were observed with SEM to check for bacterial adhesion on the surface. Before SEM observation, disks with adhered bacteria were dried in ambient environment and sputter coated with gold/palladium. SEM images were recorded with a LEO 1550 SEM (Zeiss, Oberkochen, Germany) using the in-lens detector and 10 kV acceleration voltage.

Three disks from each of the control disks, the post-UV dry disks and the post-UV wet disks were assessed for viability after the adhesion culturing process. The amount of viable bacteria on each disk was quantified using the metabolic assay incorporating resazurin. Each disk was placed upside-down in a well of a 48-well plate with 500 µL sterile PBS. The plate was placed in an ultrasonic bath for 1 minute to detach the bacteria from the surface. Afterwards, 100 µL of this bacterial suspension was transferred to a well with 900 µL BHI broth with resazurin (1.25 µg/mL) in a 48-well plate. A dilution series of *S. epidermidis* with known bacterial concentrations was performed in parallel to provide a standard curve. Both the test sample and calibration assays were incubated at 37°C for 4 hours and the production of resorufin was measured with fluorescent multiplate reader (excitation at 530 nm, emission at 590 nm). The number of bacteria in each test sample well was determined with aid of the standard curve from the *S. epidermidis* calibration series.

## Results

### Resin-TiO_2_ nanocomposite

**Figure 1:**
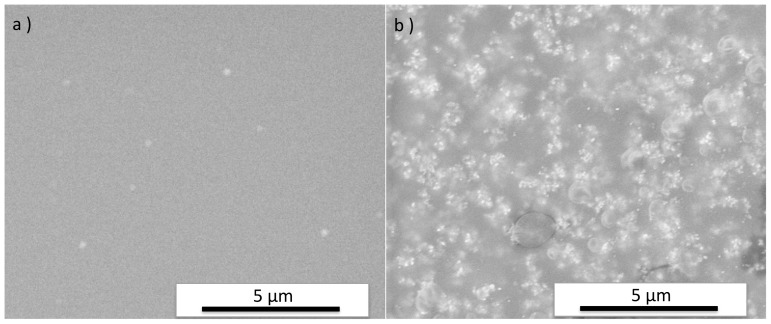
SEM images of sample disks. a) pure resin disk without addition of TiO_2_ and b) resin-TiO_2_ nanocomposite disk.

**Figure 2:**
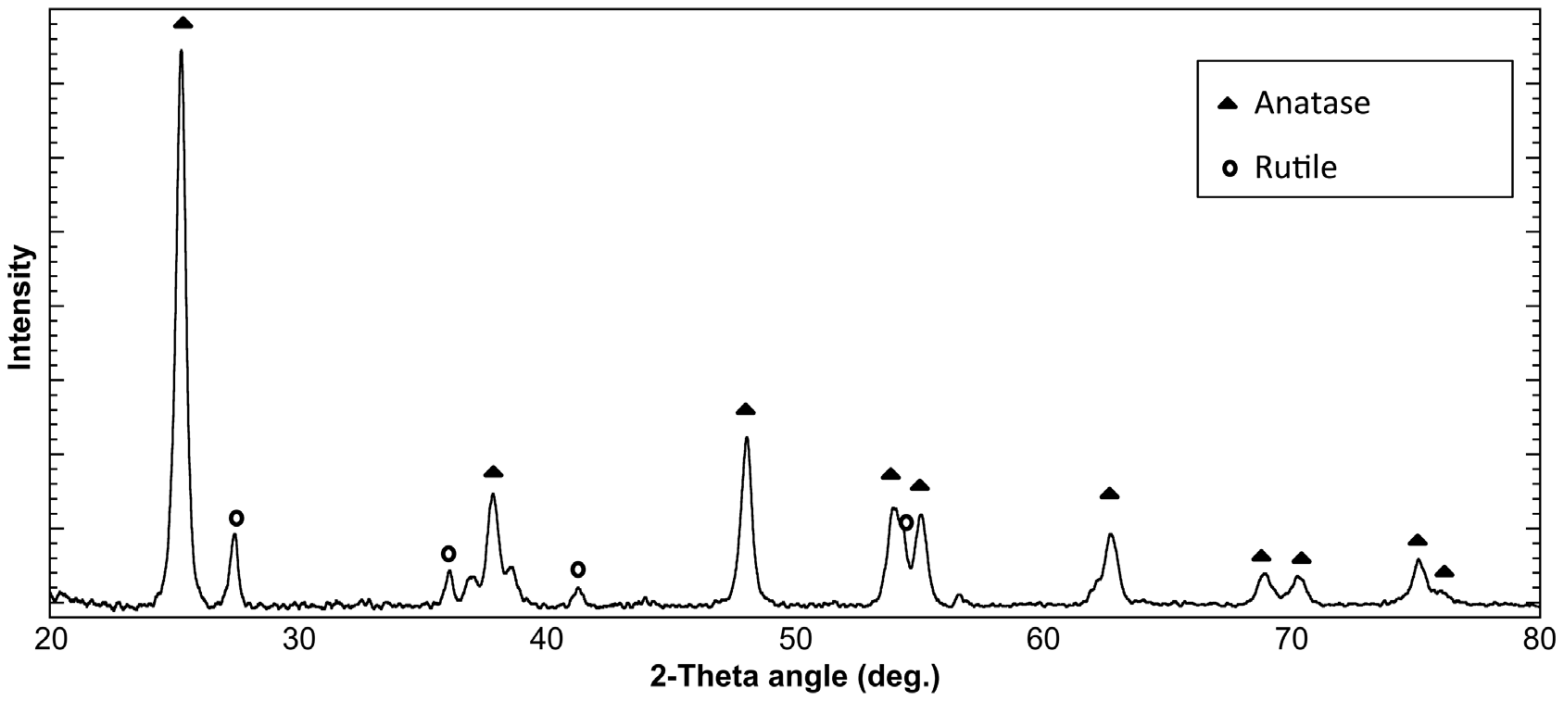
XRD pattern of a resin-TiO_2_ disk. Diffraction peaks pertaining to the anatase and rutile phases of TiO_2_ are indicated.

Figure 1 shows SEM images of the surfaces of a pure resin disk and a resin-TiO_2_ nanocomposite disk. The TiO_2_ nanoparticles encased in the resin matrix can be observed in the image of the resin-TiO_2_ nanocomposite. An XRD pattern of the resin-TiO_2_ disk (Figure 2) shows distinct diffraction peaks of anatase and rutile phases of TiO_2_, which are attributed to the P25 nanoparticles encased in the resin.

### Post-UV antibacterial tests

**Figure 3:**
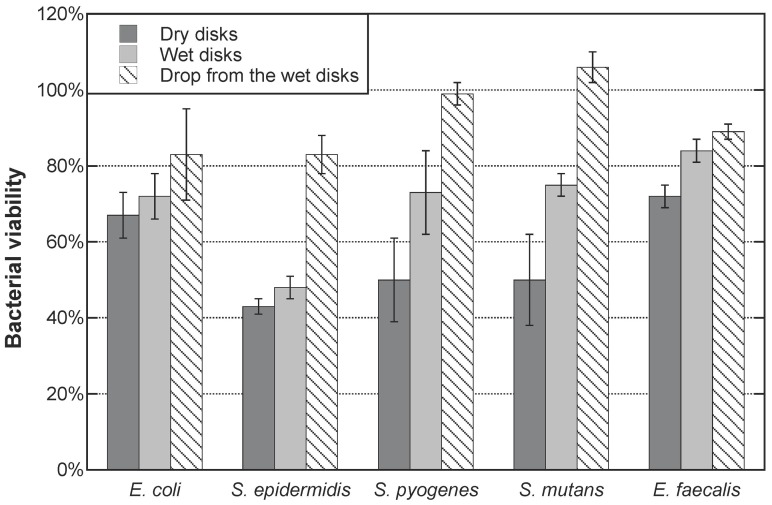
Post-UV antibacterial effects against five bacterial strains. Standard deviations are derived from three measurements. 100% viability corresponds to the bacterial viability of each strain measured on the resin-TiO_2_ disks without UV pre-treatment.

Figure 3 shows the post-UV antibacterial effect of the resin-TiO_2_ nanocomposite disks after UV irradiation in ambient condition (post-UV dry disks) and aqueous condition (post-UV wet disks) against five bacterial strains. The prolonged bactericidal effects of the water drops collected from the post-UV wet disks are also displayed.

The post-UV dry disks exhibited a more efficient antibacterial effect than the post-UV wet disks. In the tests with *E. coli, S. epidermidis*, *S. pyogenes*, *S. mutans* and *E. faecalis*, the post-UV dry disks achieved a 33%, 57%, 50%, 50% and 27% reduction of bacterial viability, respectively, compared to the bacterial viability measured on the resin-TiO_2_ control disks for each strain. The corresponding reduction of bacterial viability caused by the post-UV wet disks was 27%, 52%, 27%, 25% and 16%. The water drops extracted from the post-UV wet disks provided less bactericidal effect than the post-UV disks. The water drops reduced the viability of *E. coli*, *S. epidermidis* and *E. faecalis* by 17%, 17% and 11% respectively, but did not reduce the viability of *S. pyogenes* and *S. mutans*.

These results show that susceptibilities of the five bacterial strains to the post-UV antibacterial effect vary. Among the five species, *S. epidermidis* is the most sensitive to both dry and wet post-UV disks. *S. pyogenes* and *S. mutans* are relatively more sensitive to post-UV dry disks than to post-UV wet disks. Finally, *E. coli* and *E. faecalis* are the least affected by the post-UV disks compared to other strains.

**Figure 4:**
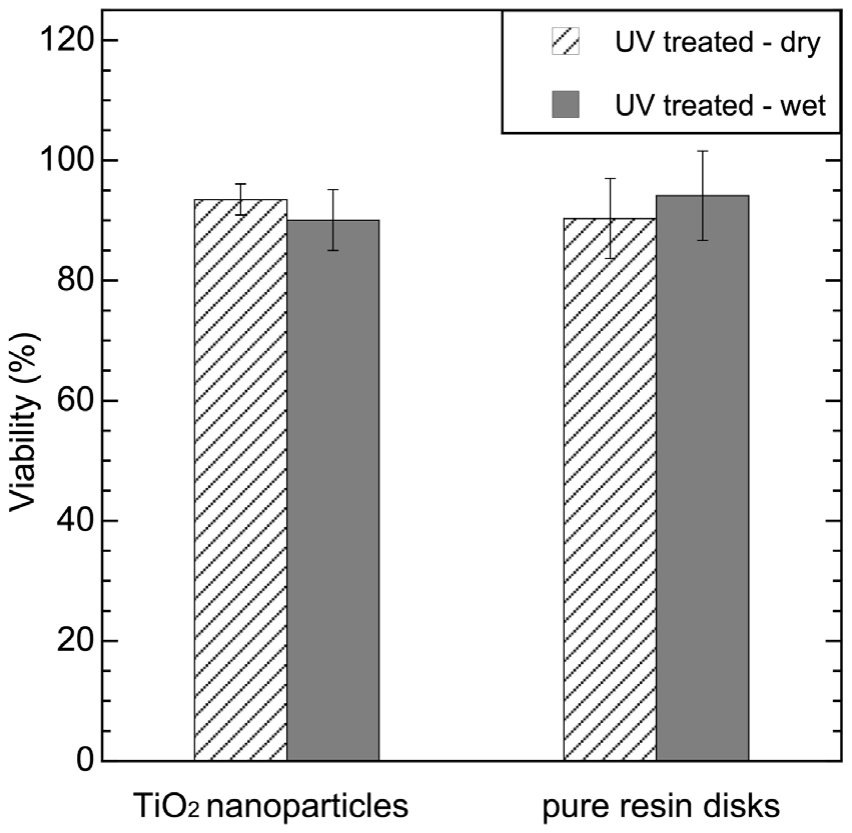
Post-UV antibacterial effects of P25 TiO_2_ nanoparticles and pure resin disks against *S. epidermidis*. 100% viability corresponds to the bacterial viability of *S. epidermidis* measured on the corresponding samples without UV pre-treatment.

Neither the pure resin disks nor the TiO_2_ nanoparticles exhibited a significant post-UV antibacterial effect as shown in Figure 4. This indicates that the post-UV antibacterial effect requires a combination of TiO_2_ nanoparticles and resin.

### Bacterial adhesion tests

**Figure 5:**
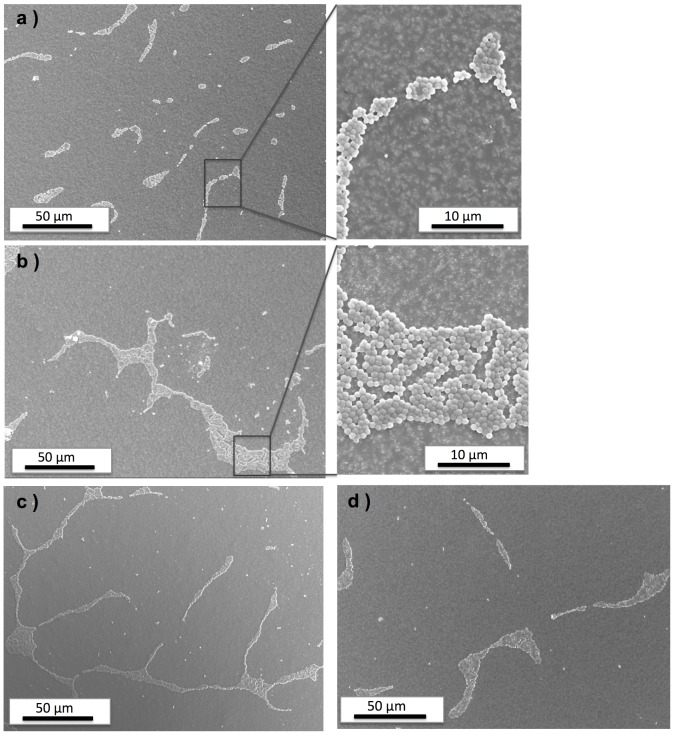
SEM images of resin-TiO_2_ disks after *S. epidermidis* adhesion culturing. a) disk from Control Group 1 without UV pre-treatment; b) post-UV dry disk; c) post-UV wet disk; d) disk from Control Group 2 without UV pre-treatment but irradiated with UV during adhesion culturing.

Figure 5 displays SEM images of the surfaces of four types of disks after the adhesion culturing: a resin-TiO_2_ disk without UV pre-treatment (Control Group 1, panel a), post-UV dry disk (panel b), post-UV wet disk (panel c) and a resin-TiO_2_ disk without UV pre-treatment but irradiated with UV during adhesion culturing (Control Group 2, panel d). In all disks a similar pattern of *S. epidermidis* adhesion was observed. Under a shear force during culturing, bacteria are prone to attach to each other while adhering to the resin-TiO_2_ surface rather than dispersing separately on the surface. Qualitatively, no significant differences could be observed in the adhesion tendencies of *S. epidermidis* on the four types of disks.

**Figure 6:**
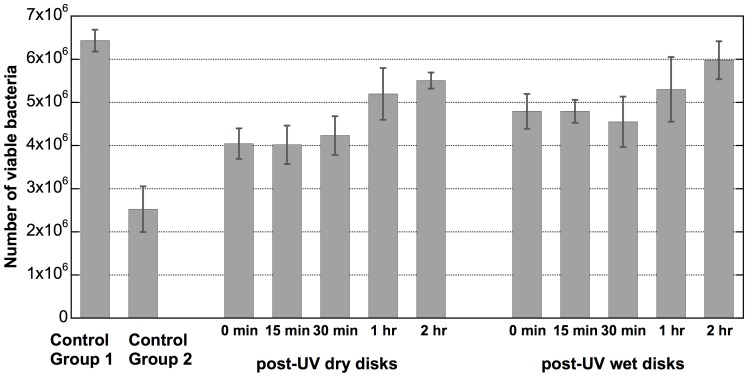
Viability of adhered bacteria on resin-TiO_2_ disks. Post-UV dry and post-UV wet disks were cultured following a delay period of 0, 15, 30, 60 or 120 minutes after the UV pre-treatment. Control Group 1 are resin-TiO_2_ disks without UV pre-treatment and Control Group 2 are resin-TiO_2_ disks without UV pre-treatment, but illuminated during the adhesion culturing. Standard deviations are derived from three measurements.

The viability of adhered *S. epidermidis* bacteria on the four groups of resin-TiO_2_ disks is shown in Figure 6. The viability of the bacteria adhered to the resin-TiO_2_ disks was derived from the metabolic activity assays based on measurements of resazurin conversion. The viability of Control Group 2 shows that the photocatalytic antibacterial effect resulting from the UV irradiation during the culturing produced the greatest reduction in viability in adhered bacteria (61%) compared to Control Group 1 in which no antibacterial effect can be assumed. Note that the total UV dose irradiated on Control Group 2 disks (10 mW/cm^2^ for 1 h) is equivalent to the UV dose used in the UV pre-treatment of the post-UV dry and post-UV wet disks. The post-UV dry disks and post-UV wet disks showed a 37% and 26% reduction, respectively, of viable adhered bacteria compared to Control Group 1.

From Fig. 6 we can also observe a diminishing post-UV antibacterial effect over time. A similar degree of bactericidal effect was observed for both the post-UV dry and post-UV wet disks that were cultured within 30 min following the UV pre-treatment. However, this effect diminished if the delay period following the UV pre-treatment was increased. For example, the viability of adhered bacteria on the post-UV wet disks that were cultured 120 min after the UV pre-treatment showed the same viability as disks that did not receive a UV pre-treatment (i.e., Control Group 1 disks).

**Figure 7:**
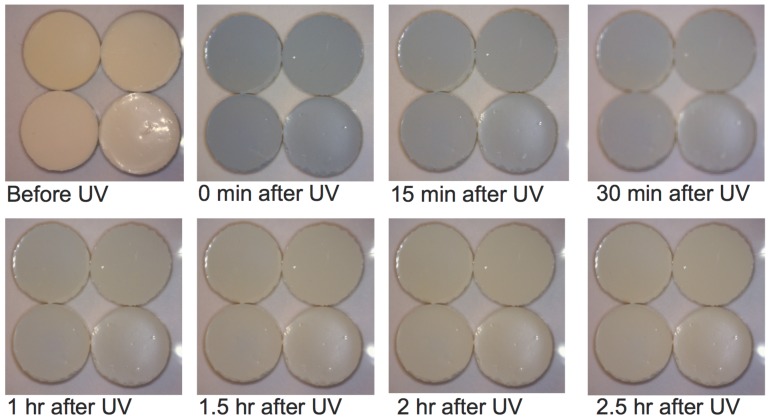
Appearance of the resin-TiO_2_ disks before and after one hour of UV irradiation under ambient conditions at 10 mW/cm^2^.

A visual indication of a diminishing post-UV effect can be observed in Figure 7 where the appearance of resin-TiO_2_ disks both before and following a UV treatment of 1 h at 10 mW/cm^2^ is displayed. During the UV irradiation, the color of the disks changed from white to blue. When the UV light was removed, the blue color of the disks faded away gradually and eventually changed back to white.

## Discussion

Whereas existing literature indicates only an immediate antibacterial effect associated with the photocatalytic process [4], [13], the results presented in this study clearly reveal a prolonged antibacterial effect after the cessation of UV irradiation of the resin-based TiO_2_ nanocomposites under study. With all bacterial strains tested, the antibacterial effect of the post-UV dry disks was larger than that of the post-UV wet disks. One possible explanation for this is that the UV pre-treatment may have resulted in different surface modifications on the two types of disks and, thus, may have influenced how the bacterial suspensions placed on the disks interacted with the surface. For example, a more hydrophilic surface may have allowed the bacteria to come closer to the surface and, hence, resulted in a greater antibacterial effect than on a more hydrophobic surface. Another explanation could be that the post-UV wet disks simply had a diminished antibacterial capacity due to the release of ROS into the water drop during the UV pre-treatment. The antibacterial effect provided by the water drop, as shown in Fig. 3, can be attributed to the presence of ROS generated during the UV pre-treatment. Hydrogen peroxide is the most likely ROS due to the relatively short lifetimes of the •OH and O_2_^•—^ radicals.

From Fig. 3 we can also observe that the degree of inactivation varied depending on the bacterial strain. The ability of bacteria to withstand attack from ROS depends on their innate characteristics, such as, cell wall and cell membrane structure and thickness as well as ROS-scavenging systems [4], [27]. Bacteria, like many other organisms, have ROS-scavenging systems to protect themselves from oxidative stress, like intracellular ROS produced during aerobic metabolism and extracellular ROS due to host defense response or environmental stresses [28], [29]. For example, *E. coli* and *Staphylococcus* are catalase- and SOD-positive, which means they have the capability of scavenging H_2_O_2_ and superoxide radicals (O_2_^•—^), respectively [30]. *Streptococcus* and *Enterococcus* are catalase-negative and SOD-positive [31], [32]. Therefore, all strains utilized in this work have a certain level of resistance to ROS. However, in photocatalytic disinfection, large amounts of ROS are produced that attack the bacteria from outside of the cell wall. Such an excess of ROS would also likely overwhelm the ROS-scavenging systems. In this work, *E. coli* (the only Gram-negative strain in the tests) and *E. faecalis* exhibited the greatest resistance to the post-UV antibacterial effect of the disks while *S. epidermidis* was the most susceptible. Both of the *Streptococcus* bacteria, *S. pyogenes* and *S. mutans* showed very similar behavior in response to both types of post-UV disks and the water drop extracted from the post-UV wet disk. *S. mutans* are known to be able to survive in environments with relatively high concentrations of H_2_O_2_ since they co-exist with *Streptococcus sanguinis*, a H_2_O_2_ producing bacteria in dental biofilm [33]. This helps explain the absence of antibacterial effect of the water drop on *S. mutans*.

The bacterial adhesion testing showed that neither direct photocatalysis on the Control Group 2 disks nor the post-UV effect appeared to affect the adhesion tendencies of *S. epidermidis*. This can be seen in Fig. 5 where all disk types show approximately the same adhesion patterns and amounts of adherent bacteria. We cannot expect that the antibacterial effects will remove the bacteria as it is known that decomposition of bacteria due to photocatalysis takes significantly longer time than inactivation of the bacteria. For example, previous studies have shown that it can take as much as a week of UV irradiation to completely remove *E. coli* from a TiO_2_ surface [34]. On the other hand, the viability of the adhered bacteria was significantly reduced on both the Control Group 2 disks and the post-UV disks. Again we observe a slightly greater antibacterial effect from the post-UV dry disks compared to the post-UV wet disks. The fact that direct UV irradiation of the Control Group 2 disks caused an even higher degree of inactivation is perhaps not surprising as the bacteria would be affected by ROS generated both during and post UV irradiation (note that the UV dose with the Control Group 2 disks was equivalent to the UV pre-treatment dose of the post-UV disks). Additionally, bacterial viability may have been further reduced by the antibacterial effect of UV alone in the Control Group 2 tests.

An interesting observation in the viability measurements of the adherent bacteria is the diminishing of the post-UV effect when the *S. epidermidis* was cultured more than 30 min after the end of the UV pre-treatment. Another indication of this diminishing effect with time can be seen in Fig. 7 in which we can see a change in color of the disks with time after a UV pre-treatment. In fact, most of the blue color in the disks disappears after 30 min. This blue color can be attributed to the presence of Ti^III^ sites caused by photo-produced electrons that are trapped at the surface of the TiO_2_ nanoparticles [35], [36]. It is likely that the resin material encasing the nanoparticles slows the diffusion of oxygen to the surface of the nanoparticles that then react with the Ti^III^ sites to create the O_2_^•—^ radical. This could explain the prolonged antibacterial effect of the post-UV disk.

The post-UV effect of the resin-TiO_2_ nanocomposite has foreseeable benefits for biomaterial and biomedical device applications. For example, if the nanocomposite is used as a dental material, not only can UV irradiation be used to inactivate infectious bacteria, the post-UV effect can help reduce contamination of the surface after the treatment. Similarly, if such a material is used as a coating for implants, the post-UV effect can help prevent contamination of the surface after sterilization of the surface with UV/photocatalysis.

## Conclusion

In this work, we presented a novel property of a resin-TiO_2_ nanocomposite in which an antibacterial effect is shown to be present for a significant time following UV irradiation. This post-UV effect of the resin-TiO_2_ disks was shown to be effective against five bacterial strains, including *E. Coli*, *S. epidermidis*, *S. pyogenes*, *S. mutans and E. faecalis*. The post-UV antibacterial effect was also tested on the tendency for *S. epidermidis* adhesion on the disk surface. Although the ability of the bacteria to adhere to the surface was not affected by the post-UV disks, the viability of the adhered bacteria was reduced by 37% compared to disks that did not receive the UV pre-treatment. Additionally, the post-UV antibacterial effect remained for at least 30 minutes after the UV treatment. The prolonged antibacterial effect could help reduce the chance of contamination by pathogenic microbes after the cessation of UV irradiation and is therefore promising for applications in biomaterials or implant coatings.
